# Life Trajectories Through the COVID-19 Pandemic: A Repeated Measures Diary Survey Dataset From 2020-2021

**DOI:** 10.3389/fpsyg.2022.817648

**Published:** 2022-05-02

**Authors:** Eric Allen Jensen, Axel Pfleger, Lars Lorenz, Aaron Michael Jensen, Brady Wagoner, Meike Watzlawik, Lisa Herbig

**Affiliations:** ^1^Institute for Methods Innovation, Dublin, Ireland; ^2^Institute for Psychological Research at the SFU Berlin e.V., Berlin, Germany; ^3^Qualia Analytics, Dublin, Ireland; ^4^Department of Communication and Psychology, Aalborg University, Aalborg, Denmark; ^5^Bjørknes Høyskole, Oslo, Norway

**Keywords:** COVID-19, vaccination, conspiracy theories, risk perception, information behavior

## Introduction

Many psychological, sociological and communication challenges have emerged or become attenuated during the COVID-19 pandemic. To understand these challenges, we need to gain an in-depth understanding of the role and perspective of individuals as they coped with this long-running global crisis. For example, while the macro-level life-and-death consequences of (non-)compliance with mitigation measures aimed at reducing the spread of the virus have already been shown in the medical literature, accurately accounting for the temporal unfolding of within-individual psycho-social and information-seeking factors. Taking multiple snapshots of these multidimensional factors in a wider time window may contribute to better explanations of such behavior, yet requires specialized research methods. Goisis and Moroni (2021) have provided their individual psychoanalytic accounts of the COVID-19 pandemic via personal diaries, and Munyikwa (2020) similarly documented their own personal pandemic experiences as an anthropologist. Taking a more outward perspective, Ibrahim et al. (2021) examined the feasibility of evaluating the psychosocial effects of virtually guided exercise on elderly citizens through a repeated measures study during the first COVID-19 wave. To our knowledge, however, there are no datasets nor other literature yet published that incorporate comprehensive methods investigating the social dimensions of, and public attitudes related to, the pandemic over an extended period. To foster evidence-based responses to the challenges posed by this public health crisis (e.g., see Jensen and Gerber, 2020), we designed a diary survey for linked sequential measurements sufficiently frequent to allow understanding in granular detail the pandemic-related perspectives and experiences of individual members of the public in Germany.

These intensive longitudinal data on German public responses to the COVID-19 pandemic over 11 months show stability and change in opinions, outlooks and coping responses during this critical period in history. This study design enables exploration of phenomena that may appear stable in a cross-sectional analysis, yet may in fact be variable when multiple snapshots are taken and considered in a wider time-window (Van de Ven and Sminia, 2012; Roe, 2014). In contrast to more commonly developed retrospective accounts (Wagoner and Jensen, 2015), the data in this study were collected via a biweekly diary survey as part of the Viral Communication project, which investigated the social and ethical dimensions of the COVID-19 pandemic in Germany.

## Methods

With the diary survey, we collected paired sample response data in Germany between November 2020 and September 2021, using a repeated measures design conducted across a maximum of 18 measurement points. The diary survey was conducted in parallel with a longer repeated measures survey with an overlapping, much larger sample that took place at three measurements. Some survey items were identical between both surveys so that in total, a maximum of 21 measurement points were possible if respondents participated in all steps for both surveys.

Both surveys were part of the Viral Communication project. This project involved an interconnected set of qualitative and quantitative data collection and analysis (viralcomm.info). Standard good practice in social research for informed consent, data collection and management and anonymization methods were employed (see Jensen and Laurie, 2016). All research protocols and procedures for the Viral Communication project were reviewed and approved by the Ethics Committee of Sigmund Freud University.

### Data Collection

The research used a software solution designed for paired samples with matching between responses at the individual level, as well as automated email invitations and reminders for the questionnaires. All data collection used digital software for secure online and GDPR-compliant data collection and management provided by the research technology company Qualia Analytics. The dataset described in this paper involves data from two related surveys, namely a repeated measures survey (hereinafter referred to as main survey) with three stages and a larger sample (October-December 2020, March 2021, and August-September 2021) as well as a diary survey with 18 stages. For the main survey, a representative probability-based sample was acquired through a postal recruitment system (for a full description, see Jensen et al., 2021). At the end of the first iteration of the main survey, respondents were able to opt in for additional studies, inter alia, the diary survey. Two weeks after their response to the main survey, consenting respondents were sent email invitations to participate in the diary survey. Following their first completed diary survey stage, reminders for the next survey stages were sent in two-week intervals from the time they completed their last diary survey stage. Through the automatic matching of responses across the surveys using the specialized software for repeated-measures studies described above, we avoided the need to ask respondents the same socio-demographic questions in each survey stage.

From the main survey respondents (*N* = 1,480), *N* = 133 completed the first diary survey stage (*p*_woman_ = 54%; *M*_age_ = 48.6, *SD* = 14.5), resulting in an initial response rate of about 9% for the diary survey. From the second stage on, the attrition rate (i.e., the proportion of respondents who discontinue their repeated participation in the survey) constantly remained under 10% (see Table 1). However, only 51% of those who initially participated in the diary survey completed the 18^th^ diary survey stage, leaving a final *N* = 68 (*p*_woman_ = 61%; *M*_age_ = 49.4, *SD* = 11.7).

**Table 1:** Attrition rate for each diary stage respective to each previous diary stage, including the corresponding portion of the initial diary survey sample.

**Diary stage**	***N***	**Attrition rate**	**Portion of original sample**
1	133	-	100%
2	122	8%	92%
3	120	2%	90%
4	118	2%	89%
5	114	3%	86%
6	108	5%	81%
7	105	3%	79%
8	101	4%	76%
9	99	2%	74%
10	96	3%	72%
11	92	4%	69%
12	89	3%	67%
13	88	1%	66%
14	87	1%	65%
15	84	3%	63%
16	78	7%	59%
17	71	9%	53%
18	68	4%	51%

### Survey Instrument

The diary survey had mostly closed-ended items such as single-response questions, Likert-type scales, and semantic differentials. We limited the scope of the questionnaire to research topics of the highest relevance for this kind of intensive repeated-measures research in order to reduce the strain of repeated participation and therefore maximize the retention rate for the survey. For the same practical purpose, we split the questionnaire into a main section and an optional section. Mean completion time per diary survey stage was 8.5 min. The proportion of respondents who continued to the optional second section of the survey ranged from 87 to 99%.

The first two survey items investigated respondents' COVID-19 infection history within their household. The question, “Have you ever had, or thought you might have, the Coronavirus (COVID-19)?” was answered via a single-response item with the options *Yes, No*, and *Unsure*. If respondents selected Yes, the follow-up question, “Have you been tested for COVID-19?” was asked. The single-response options here were Yes, and I tested positive (confirmed COVID-19), *Yes, but I tested negative (no COVID-19)*, and *No, I have not been tested*. These two survey items were employed both in the diary survey and the first main survey iteration.

From here until the opt-in optional section, all survey items were used across all surveys. In this section, we asked respondents whether they had “faced any challenges in accessing useful pandemic-related information in the last 2 weeks,” using a single-response item. The response options were *Yes, No*, and *Unsure*.

The next statement, “In the last 2 weeks, I have taken the following measures to protect myself and others from Coronavirus (COVID-19) infection,” was followed by a set of 7-point Likert-type items investigating how often respondents adhered to infection mitigation behaviors. The ordinal response options were: *Never, Rarely, Occasionally, Sometimes, Frequently, Usually* and *Always*, plus *Not applicable / No opinion*. The first of these items was “Maintaining a distance of at least 1.5 meters from other people.” At each diary survey stage, one of two items was shown at random as the second item: “Avoiding foreigners,” or “Using hand sanitiser or washing hands after visiting public spaces”. The “avoiding foreigners” item was designed to assess the intersection between pandemic-inspired public health concerns and xenophobia. The same randomized structure applied to the next two items in the diary survey: “Wearing a protective mask where mandatory” or “Using the Corona-Warn-App” (i.e., the main track and trace app for Germany).

An additional set of Likert-type items was introduced, asking respondents to indicate their level of agreement with the following statements: “I am concerned about the health of my family/friends,” and “I am concerned about my own health.” The response options included the 7-point agreement scale, *Strongly Disagree, Disagree, Somewhat Disagree*, Neutral, Somewhat Agree, Agree, *Strongly Agree*, as well as *Not applicable/No opinion*.

Respondents' trust in key governmental and scientific representatives was ascertained through another set of ordinal Likert-type items with the response options: Completely distrust, *Partially distrust, Neither distrust nor trust, Partially trust, Completely trust*, and *Not applicable / No opinion*. Of the six following items, three were displayed at random at every diary survey stage: “Christian Drosten (German virologist)” (a highly prominent scientific expert in Germany during the pandemic), “German Public Health Ministry,” “World Health Organization,” “Your state government,” and “Robert Koch Institute” (leading public health agency in Germany).

The last items in the main section of the diary survey were about support for mandatory and voluntary vaccination. Respondents were asked, “How would you feel if the following were announced as a mandatory measure by your state government?” On a 5-point Likert-type scale, respondents selected whether they would: *Strongly oppose, Oppose, Neither oppose nor support, Support*, or *Strongly support* mandatory vaccination. *Not applicable/No opinion* was included as a response option in both items. Respondents were also asked whether they would take the COVID-19 vaccine on a voluntary basis. The 5-point Likert-type scale corresponding to this question included the ordinal response options *Definitely not, Probably not, Maybe, Probably*, and *Definitely*. At each diary survey stage, one of the two items presented above were displayed randomly. Both items included the response option *Not applicable/No opinion*.

The remaining items were unique to the diary survey (not replicated in the linked longer survey). These were placed in the opt-in optional section. When respondents chose to answer additional questions, they were first asked about the “extent [to which they] disagree or agree with these statements” about the pandemic's impact on their lives, on the 5-point Likert-type agreement scale: “The Coronavirus (COVID-19) has had negative impacts on my life in the last 14 days,” and “The Coronavirus (COVID-19) has had positive impacts on my life in the last 14 days.”

At each diary survey stage, respondents had a 50% (randomized) chance of being asked an additional question about how they thought “the Coronavirus (COVID-19) pandemic situation will be [...] in the next 6 months.” This item was a semantic differential with *Much worse* and *Much better* at the opposite poles of the scale. Here, *Unsure* was provided as an additional response option.

To reiterate, the heavy use of randomization in the optional part of the diary survey was aimed at reducing the length of the survey to a manageable level for respondents while providing coverage of a wider range of variables than would otherwise be feasible. Randomization was also used throughout the survey design to mitigate methodological issues such as possible response bias due to question order.

## Interpreting the Dataset

The dataset was prepared by merging the main survey dataset with the diary survey dataset in IBM SPSS Statistics using respondents' unique ID, limiting the subsample to only those who contributed at least one diary survey entry. The resulting dataset is structured in wide-format and includes every quantitative variable employed in the diary survey. Due to this particular format, there are up to 21 repetitions of the same survey item. The specific survey iteration corresponding to each variable can be identified by the variable name prefix. The letters “M” and “D” indicate *main* (i.e., the longer survey that was conducted with a large number of respondents three times) and *diary* survey, respectively. The letters are followed by a number indicating the survey iteration number. For instance, all variables corresponding to the third main survey iteration will be indexed with “M3_”. The variable label will also clearly indicate the survey iteration.

We structured the dataset into multiple segments. The first segment includes relevant metadata (marked by a sole “M_” either at the beginning of the variable name or after the survey iteration prefix), and is followed by socio-demographic data. The variable “OI_AQ” indicates whether respondents continued with additional questions in the opt-in section of each survey. Variables following “OI_AQ” are opt-in questions.

It is important to note that respondents completed each diary stage at different times. This means that a particular diary stage will not have been completed within the same time frame by all respondents. In fact, some dates of completion for different diary stages might overlap. The individual dates and times of completion are indicated by the “M_COMP_DATE” variables. For repeated measures analyses, we suggest restructuring the dataset to long-format and collapsing the completion date into appropriate time categories. If needed, the data can then be restructured back to wide-format.

This diary dataset can additionally be merged with the main survey dataset (see Jensen et al., 2021) to enable analyses that involve variables which were only employed in at least one iteration of the main survey. Merging can be done based on respondents' matching unique IDs (variable name: “M_ID”). To avoid duplicate variables, we recommend that data users remove the main survey variables from the diary dataset before merging.

## Using the Dataset

The diary survey dataset described in this paper provides valuable information about the development of public perspectives and behaviors over the course of nearly one year during the COVID-19 pandemic. Variables from the main survey that were identical to the diary survey were included to provide additional data points for secondary analysis. For more complex analyses and increased flexibility, we encourage users to merge the main survey dataset (Jensen et al., 2021) with the diary dataset described in this paper. We intend for research making use of this evidence base to deliver insights that can inform current and future disaster and emergency management communication practice and policies.

The dataset is accessible on the open science publication platform Zenodo as an SPSS file: https://doi.org/10.5281/zenodo.5702833. The data are fully anonymized and cleaned.

## Data Availability Statement

The original contributions presented in the study are included in the article/supplementary materials, further inquiries can be directed to the corresponding author/s.

## Ethics Statement

The studies involving human participants were reviewed and approved by Ethics Commission of the Sigmund Freud University. The patients/participants provided their written informed consent to participate in this study.

## Author Contributions

AP and LL set up the survey system. Data collection was conceptualized by EJ, BW, and MW and implemented by LL. AP performed the data management and wrote up the article. EJ and AJ did final editing. All authors contributed to the survey design. All authors contributed to the article and approved the submitted version.

## Funding

The research presented in this paper was funded by the German Federal Ministry of Education and Research under grant agreement 01KI20500.

## Conflict of Interest

AP, LL, and AJ were employed by Qualia Analytics. The remaining authors declare that the research was conducted in the absence of any commercial or financial relationships that could be construed as a potential conflict of interest.

## Publisher's Note

All claims expressed in this article are solely those of the authors and do not necessarily represent those of their affiliated organizations, or those of the publisher, the editors and the reviewers. Any product that may be evaluated in this article, or claim that may be made by its manufacturer, is not guaranteed or endorsed by the publisher.
